# Myopathologic trajectory in Duchenne muscular dystrophy (DMD) reveals lack of regeneration due to senescence in satellite cells

**DOI:** 10.1186/s40478-023-01657-z

**Published:** 2023-10-19

**Authors:** Nastasia Cardone, Valentina Taglietti, Serena Baratto, Kaouthar Kefi, Baptiste Periou, Ciryl Gitiaux, Christine Barnerias, Peggy Lafuste, France Leturcq Pharm, Juliette Nectoux Pharm, Chiara Panicucci, Isabelle Desguerre, Claudio Bruno, François-Jerome Authier, Chiara Fiorillo, Frederic Relaix, Edoardo Malfatti

**Affiliations:** 1 Univ Paris Est Creteil, INSERM, IMRB, 94010 Creteil, France; 2 Centre of Translational and Experimental Myology, IRCCS Istituto Giannina Gaslini, Genoa, Italy; 3 APHP, Filnemus, EuroNMD, Centre de Référence de Pathologie Neuromusculaire Nord-Est-Ile-de-France, Henri Mondor Hospital, Paris, France; 4 Neurophysiologie clinique pédiatrique, Centre de référence des maladies neuromusculaires Hôpital universitaire Necker-Enfants Malades-Paris, Centre de Référence de Pathologie Neuromusculaire Nord-Est-Ile-de-France, Henri Mondor Hospital, Université Paris Est, U955 INSERM, IMRB, APHP, Creteil, France; 5 Reference Center for Neuromuscular Disorders, Filnemus, EuroNMD, Assistance Publique-Hôpitaux de Paris (APHP) Necker Enfants Malades Hospital, Paris, France; 6 Service de Médecine Génomique, Maladies de Système et d’Organe - Fédération de Génétique et de Médecine Génomique, DMU BioPhyGen, APHP Centre-Université Paris Cité - Hôpital Cochin, Paris, France; 7 Department of Neuroscience, Rehabilitation, Ophthalmology, Genetics, Maternal and Child Health-DINOGMI, University of Genova, Genoa, Italy; 8 Child Neuropsychiatry, IRCCS Istituto Giannina Gaslini, Genoa, Italy

**Keywords:** Duchenne muscular dystrophy, Fibrosis, FAPs, Muscle regeneration, Cellular senescence

## Abstract

Duchenne muscular dystrophy (DMD) is a devastating X-linked muscular disease, caused by mutations in the *DMD* gene encoding Dystrophin and affecting 1:5000 boys worldwide. Lack of Dystrophin leads to progressive muscle wasting and degeneration resulting in cardiorespiratory failure. Despite the absence of a definitive cure, innovative therapeutic avenues are emerging. Myopathologic studies are important to further understand the biological mechanisms of the disease and to identify histopathologic benchmarks for clinical evaluations. We conducted a myopathologic analysis on twenty-four muscle biopsies from DMD patients, with particular emphasis on regeneration, fibro-adipogenic progenitors and muscle stem cells behavior. We describe an increase in content of fibro-adipogenic progenitors, central orchestrators of fibrotic progression and lipid deposition, concurrently with a decline in muscle regenerative capacity. This regenerative impairment strongly correlates with compromised activation and expansion of muscle stem cells. Furthermore, our study uncovers an early acquisition of a senescence phenotype by DMD-afflicted muscle stem cells. Here we describe the myopathologic trajectory intrinsic to DMD and establish muscle stem cell senescence as a pivotal readout for future therapeutic interventions.

**Supplementary Information:**

The online version contains supplementary material available at 10.1186/s40478-023-01657-z.

## Introduction

Duchenne muscular dystrophy (DMD, OMIM#310,200) is a rare, devastating X-linked disease affecting approximately 1:5000 boys. DMD is caused by out-of-frame mutations in the *DMD* gene that abolish the production of Dystrophin protein [10]. In contrast, in-frame mutations of the *DMD* gene lead to the synthesis of a partially truncated and functional protein which is associated with a milder phenotype known as Becker Muscular Dystrophy [18]. DMD boys are diagnosed at around three years of age since they present with stereotyped walking and motor difficulties [3, 23]. Corticosteroids therapy has definitely modified the progression of the disease, improving muscle strength, delaying the loss of ambulation and the decline in respiratory and cardiac functions [35]. Nevertheless, there is no treatment at present for DMD patients who currently exhibit a median lifespan of up to 25 years [6]. Currently, genetic investigations serve as the primary diagnostic modality for DMD, impeding the accessibility of muscle biopsy specimens essential for comprehensive myopathologic analyses [4, 13, 45]. Nevertheless, muscle biopsies retain their significance not only in detecting cryptic variants but also for RNA and protein investigations. Moreover, they play a crucial role in clinical trials, enabling the monitoring of muscular responses to innovative therapeutic strategies like gene therapies. [16, 21, 24, 25]. To date there are only a few studies, performed in relatively small series of DMD patients, that tracked the myopathologic trajectory of DMD [1, 8, 29]. Furthermore, new cellular components have emerged as crucial in the trajectory of DMD advancement, including muscle stem cells (MuSCs) and fibro-adipogenic progenitors (FAPs) [9, 11, 30, 32, 38–40, 42]. Indeed, DMD is caused by the lack of Dystrophin, an essential cytoskeletal component of muscle fibers. The absence of Dystrophin not only causes myofiber fragility but also triggers cycles of muscle degeneration and regeneration with eventual loss of muscle repair and replacement of the muscle tissue by fibrosis and adipocyte infiltration [7, 44]. The ability of muscle to regenerate relies on muscle stem cells, which activate and proliferate into committed myoblasts after muscle damage, and eventually differentiate into newly generated myofibers, or maintain the stem cell pool by self-renewing [30, 41]. Muscle stem cell functions in regeneration are tightly regulated by fibro-adipogenic progenitors (FAPs) that are also the main source of fibrosis and fatty replacement in diseased muscles [20, 33, 43]. Interestingly, compromised tissue regeneration by MuSCs and high extra-cellular matrix protein production by FAPs are hallmarks of DMD [9, 11, 38–40]. Nonetheless, how these two cell populations operate within distinct stages of DMD human tissues remains poorly understood and tracking their features holds promise as innovative markers for tracking the progression of the disease. Here we analyzed a cohort of twenty-four muscle biopsies from genetically confirmed DMD boys and compared them to twenty-seven histologically normal type and age matched muscles. We yield valuable insights into myopathologic disease trajectory describing fiber damage, inflammation, fibrosis, fat deposition and mechanisms responsible for defective muscle repair, which may serve as valuable readout in future research that seeks to evaluate the efficacy of any therapeutic interventions on muscle tissue.

## Materials and methods

### Patients

We analyzed twenty-four open muscle biopsies from DMD boys. Age at muscle biopsy, biopsied muscle and genetics are reported in Table 1. Twenty-seven age-matched muscle biopsies reported as histologically normal by three certified histopathologists (EM, CF, and FJA) were used as controls (Table 2). We classified patients into five groups according to the age at muscle biopsy: 1–2 years (y), 3–4 y, 5–6 y, 7–8 y and more than 9 y. All the data presented in this study are in accordance with this classification. Dystrophin protein expression was assessed in all muscle biopsies and controls and confirmed absence of dystrophin in DMD patients.

**Table 1:** Data of DMD patients

Patient	Age at muscle biopsy (years)	Muscle	DMD mutation	CK levels	Steroid treatment at muscle biopsy
P 1	1	Q	del 51–54	49,450	No
P 2	1	Q	del 48–52	20,538	No
P 3	2	Q	del 48–54	11,681	No
P 4	2	Q	del 20–44	28,206	No
P 5	3	Q	ex.68; c.9953_9954del, p.Glu3318ValfsTer15	18,767	No
P 6	3	Q	dup 50	14,768	No
P 7	4	Q	del 44	28,830	No
P 8	4	D	del 45–50	12,000	No
P 9	5	D	del 48–50	nr	No
P 10	5	D	dup 12–16	15,900	No
P 11	5	Q	del 49–50	10,386	No
P 12	5	Q	del 46–47	21,458	No
P 13	6	Q	del 12–16	26,430	NO
P 14	6	Q	del 44	29,660	NO
P 15	7	Q	del 10–43	14,286	No
P 16	8	D	ex.25; c.3427C > 7, p.Gln1143*	9400	No
P 17	8	D	dup2	7395	No
P 18	8	Q	del 49–52	7392	No
P 19	9	Q	ex.15; c.1753del, p.Ile585PhefsTer22	15,196	No
P 20	15	Do	del 18–34	nr	Nr
P 21	15	Do	del 10–11	nr	Nr
P 22	17	Do	dup 10–17	nr	Nr
P 23	17	Do	dup 49	nr	Nr
P 24	18	Do	ex.35; c.4996C > T, p.(Arg1666Ter)	nr	Nr

*Del* Deletion; *Dup* Duplication; *Ex* Exon; *Q* Quadriceps; *D* Deltoid; *Do* Dorsal; *nr* Not reported

**Table 2:** Data of Ctr patients

	Age (years)	Sex	Muscle
C1	1	M	D
C2	1	M	D
C3	1	F	Q
C4	1	M	Q
C5	1	M	Q
C6	1	F	Q
C8	2	F	Q
C7	3	M	D
C9	3	M	D
C10	4	F	Q
C11	5	M	D
C12	5	M	D
C13	5	F	Q
C14	5	F	Q
C15	5	M	D
C16	6	M	Q
C17	7	M	Q
C18	7	M	D
C19	7	M	D
C20	8	M	D
C21	8	M	D
C22	9	F	Q
C23	9	F	D
C24	14	M	Q
C25	15	M	Do
C26	15	F	Do
C27	17	M	Do

*D* Deltoid; *Q* Quadricep; *Do* Dorsal

### Morphological studies

The muscles were freshly frozen in isopentane cooled with liquid nitrogen and conserved at -80 °C before the sectioning with the cryostat Leica CM3050S (Leica Biosystems) at 7 μm of thickness on Super Frost Plus slides (Thermo scientific, 10,149,870) as previously described [22]. The samples were stained using 0.1% Mayers hematoxylin (Sigma Aldrich) for a duration of 10 min, followed by immersion in 0.5% eosin (Sigma Aldrich). The sections were subsequently rinsed in distilled water and sequentially washed in 50%, 70%, 95%, and finally 100% ethanol. Afterward, they were incubated in xylene and mounted using Eurokitt. To stain sections with Picro Sirius red solution (Sigma Aldrich) the frozen tissue sections were rinsed with distilled water and incubated in Picro Sirius red solution for 25 min. Subsequently, the samples were washed with distilled water and dehydrated in 100% ethanol for 30 s before being mounted using Eurokitt. Digital photographs of each biopsy were obtained with a Zeiss AxioCam HRc linked to a Zeiss Axioplan Bright Field Microscope and processed with the Axio Vision 4.4 software (Zeiss, Germany). Histopathologic index, necrotic fibers and hypercontracted fibers were counted using Visilog image analysis software. The counting was done by applying a grid on the whole section and the events were counted only on the intersections. All the intersections represent the total number of events in the section. The histopathologic index was counted with a formula where the percentage of all the pathogenic events occurring in the muscles was divided by the total numbers of counted events [5]. The necrotic and hypercontracted fibers were normalized on the number of total events. The total fibrotic area (perimysial and endomysial) was measured with a macro-script that runs in an open access platform and was designed to quantify the percentage of the stained area in the field.

### Immunofluorescence studies

For immunofluorescence studies, the muscles were sectioned at 7 μm, permeabilized with Triton 0.5% and blocked in 10% BSA for 30 min at room temperature. Blocking was followed by an overnight incubation with primary antibodies at 4 °C. The following primary antibodies were used: DYS2 (Leica), PDGFRα (Invitrogen, PA5-16,571), Embryonic myosin heavy chain (eMHC) as a marker of newly regenerated myofibers [34] (Santa Cruz Biotechnology, sc-53091), Laminin (Sigma-Aldrich, L9393) and CD45 (BD biosciences, 555,843). Alexa fluo secondary antibodies were incubated for 45 min at room temperature after repetitive washes. For the analysis of MuSCs, slides were defrosted for approximately 20 min at room temperature, rehydrated for 5 min in PBS 1X and then fixed with 4% PFA in PBS 1X for 10 min at 4 °C. After washes in PBS 1X, slides were placed in cold Acetone:Methanol (1:1) solution for 6 min at − 20 °C and then incubated with 10% BSA blocking solution for 1 h. Primary antibodies were added to 1% BSA and incubated over-night at 4 °C. The following primary antibodies were used: Pax7 (Santa Cruz Biotechnology, sc-81648), Ki67 (Abcam, sp6 ab16667), P16 (Abcam, Ab108349), γH2AX (Abcam, ab11174) and P21 (Thermofisher, bs-10129R). After repetitive washes, slides were incubated with Alexa fluor secondary antibodies for 45 min at room temperature. Laminin staining was performed after the secondary antibody incubation, for 1 h at 37 °C using a conjugated antibody (NB300-144AF647). The number of Pax7 positive cells and double positive cells by colocalization with Pax7 were counted in at least ten acquisitions per sample and normalized to the total number of fibers. For Bodipy staining of lipid droplets [37], slides were defrosted for 20 min, hydrated in PBS 1X for 5 min, permeabilized in 0.5% Triton and then blocked in 10% BSA. After repetitive washes in PBS 1X, the slides were incubated with the primary antibody anti-Laminin 1:400 (Sigma-Aldrich, L9393) for 1 h at 37 °C. After washes, the slides were incubated with the secondary antibody 1:500 for 45 min at 37 °C and with Bodipy 1:500 (Invitrogen, D3922). All the slides were mounted with fluorescence mounting medium (Dako) after the counterstaining of the nuclei with Hoechst (Sigma-Aldrich, B2261).

### Statistical analysis

Data are presented as mean ± SEM and statistical analysis were performed using Mann–Whitney tests. Results were considered statistically significant at *p* value ≤ 0.05*. GraphPad Prism 9.0 was used to generate graphs and for statistical analysis.

## Results

**Fig. 1:**
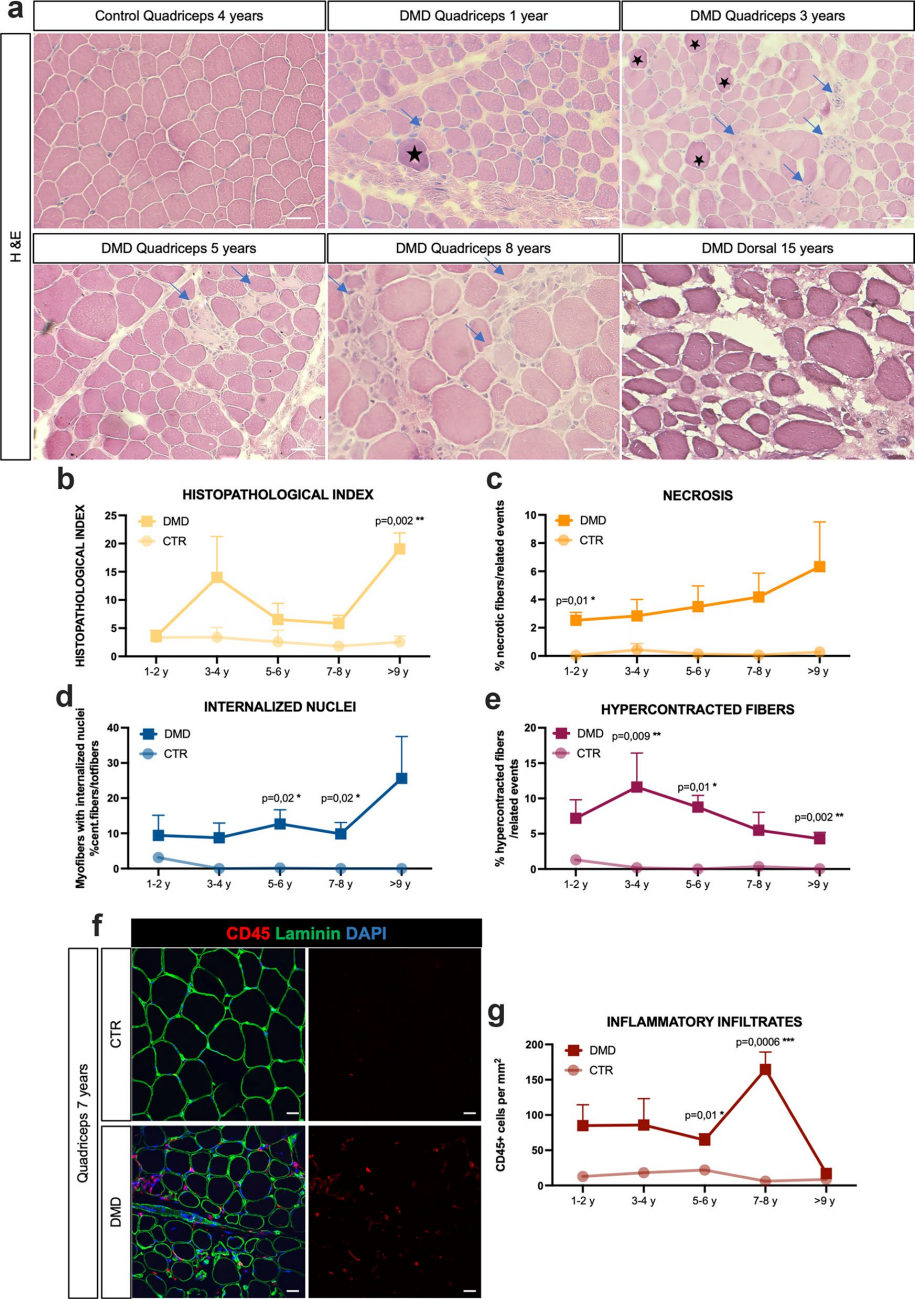
Characterization of DMD muscle phenotype. **a** Hematoxylin & Eosin (H&E) staining of Control (Ctr) and DMD muscles at different time points. Blue arrows represent necrotic fibers. Black stars represent hypercontracted fibers (scale bar = 50 μm). **b** Histopathologic index calculated on H&E stainings of Ctr and DMD biopsies at different time points. **c** Quantification of necrotic fibers of Ctr and DMD muscle from H&E. **d** Quantification of fibers with internalized nuclei on DMD and control biopsies at different time points. **e** Quantification of hypercontracted fibers on DMD and Ctr biopsies. **f** Representative immunofluorescence for CD45 (red), Laminin (green) in Ctr and DMD quadriceps at 7 years. Nuclei are counterstained with DAPI (blue) (scale bar = 20 μm). **g)** Quantification of inflammatory cells CD45-positive per mm^2^ in DMD and Ctr biopsies at different time points. Pvalues were calculated by Mann–Whitney tests comparing control and DMD groups within the same age range

DMD biopsies showed fiber size variation, and a progressive loss of muscle structure and integrity (Fig. 1a) with a higher histopathologic index compared to controls (Fig. 1b). A notable and gradual rise in the number of fibers exhibiting internalized nuclei and necrosis was observed with advancing age (Fig. 1c and d). Concurrently, the prevalence of hypercontracted fibers in DMD samples diminished with the progression of the disease (Fig. 1e). From one year of age the number of CD45 inflammatory cells was elevated in DMD muscles compared to the controls with a peak at 7–8 years, followed by a drastic decrease in those DMD patients older than 9 years (Fig. 1f and g). Interestingly, these myopathologic findings were consistent regardless of which muscle was biopsied. This first phenotypic analysis revealed the trajectories during the DMD progression of typical hallmarks of the disease such as nuclear internalizations, necrotic and hyper-contracted fibers, and inflammation.

### Muscle substitution and Fibroadipogenic progenitors (FAPs)

**Fig. 2:**
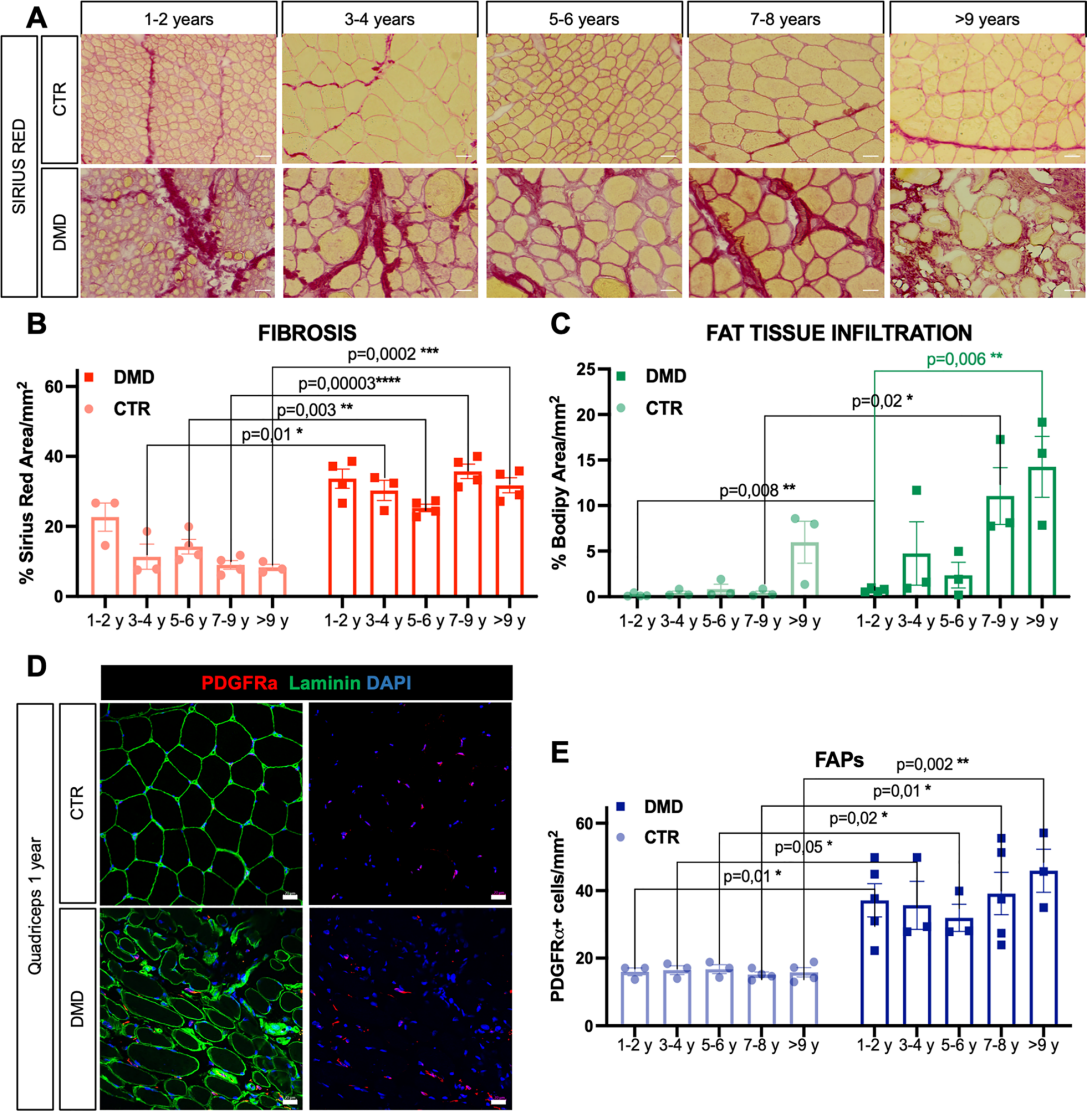
Muscle substitution and increased number of FAPs. **a** Sirius red staining on Control (Ctr) and DMD biopsies at different time points (scale bar = 50 μm). **b** Quantification of the fibrotic red-stained area in Ctr and DMD muscles on the total surface of the section (mm^2^). **c** Quantification of fat-tissue infiltration (Bodipy stained area on mm^2^) in Ctr and DMD biopsies at different time points. **d** Representative immunofluorescence for PDGFRα-positive cells (red) and Laminin (green) in Ctr and DMD quadriceps at 1 year. Nuclei were counterstained with DAPI (blue) (scale bar = 20 μm). **e** Quantification of the number of PDGFRα-positive cells on mm^2^ in Ctr and DMD biopsies at different time points. P-values were calculated by Mann–Whitney tests comparing control and DMD groups within the same age range

Sirius red staining showed a higher fibrosis in DMD muscles at all the time points (Fig. 2a and b) with statistically significant differences starting at 3–4 years of age. Fibrotic accumulation remained high without significant evolution in our DMD cohort. From 1 year old, the presence of adipocytes within the muscle was elevated, compared to control muscles (Fig. 2c). In addition, increasing levels of fatty replacement have been identified alongside the progression of the disease (Fig. 2c). Both fibrosis and fat infiltration are due to fibro-adipogenic progenitor differentiation into fibroblasts or adipocytes [43], thus we quantified the number of FAPs marked by the expression of PDGFRα [42]. At all the examined time points, there was an elevated presence of PDGFRα-positive cells within DMD muscles when compared to the control group. Moreover, no progressive increases were observed over time, consistent with the fibrotic levels observed in DMD muscles (Fig. 2d and e), suggesting a correlation between number of FAPs and fibrotic deposition.

### Impaired regeneration and satellite cells senescence acquisition with DMD progression

**Fig. 3:**
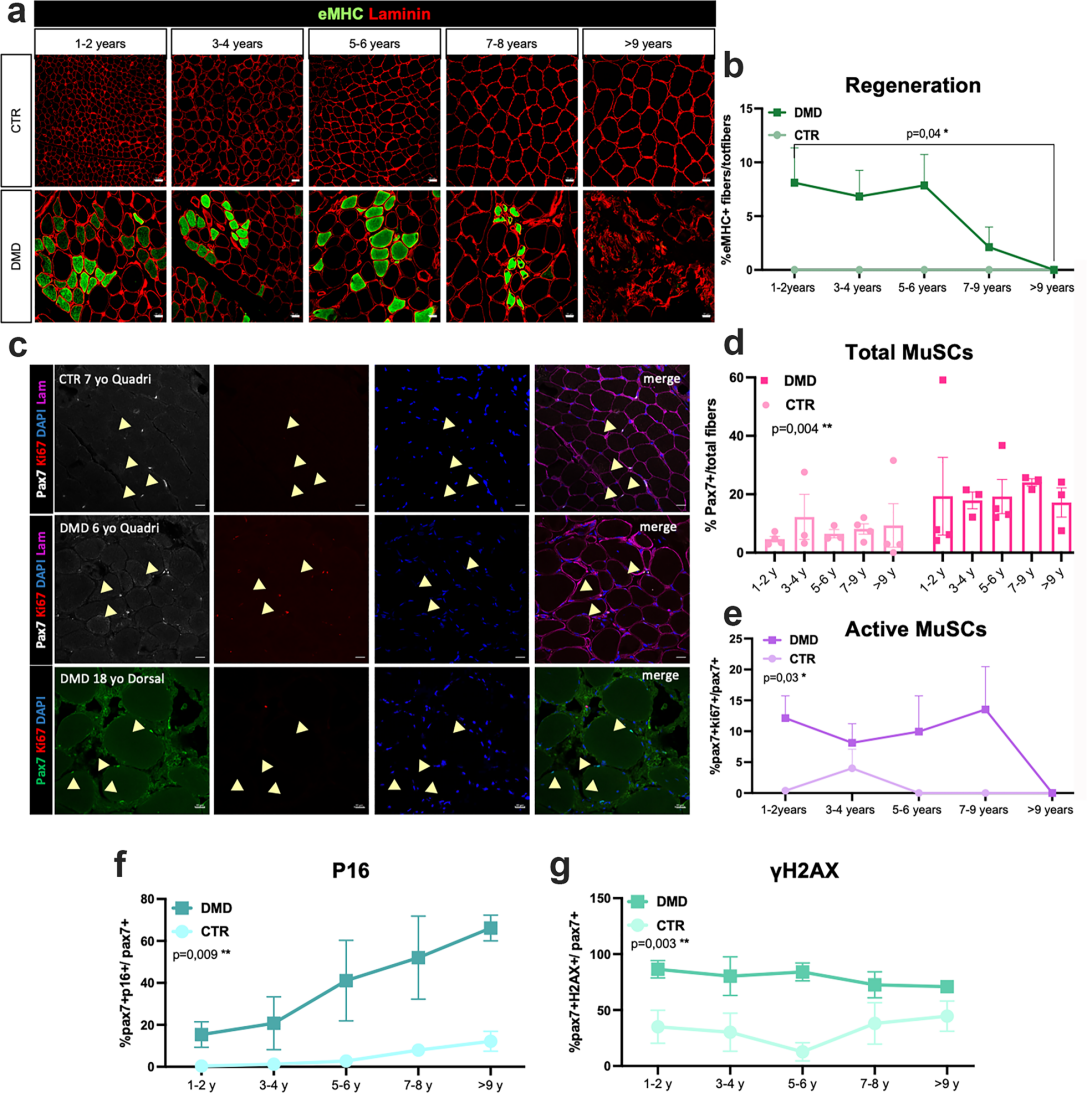
Lack of regeneration and acquisition of a SCs senescent phenotype in Duchenne muscles. **a** Co-immunostaining of regenerative fiber, eMHC-positive (green) and Laminin (red) of Ctr and DMD muscles at different time points. Scale bar = 20 μm. **b** Quantification of the number of regenerative fibers, normalised on the total amount of fibers. A *t*-test was performed between 1–2 years and > 9 years old DMD groups. **c** Representative co-immunofluorescence of Pax7, Ki67 and Laminin in Ctr Quadriceps (Quadri, 7 years), DMD (Quadri 6 years) and in DMD dorsal muscles (Dorsal, 18 years). Yellow arrows point MuSCs (Pax7+). Nuclei are counterstained with DAPI (blue) (scale bar = 20 μm). **d** Quantification of the total number of Pax7+ cells on the total amount of fibers in DMD and control muscles at different time points. **e** Quantification of the number of active MuSCs (Pax7-Ki67 double positive) on the total number of MuSCs in DMD and Ctr muscles at different age. **f** Quantification of Pax7-P16 double positive MuSCs. **g** Quantification of Pax7-γH2AX double positive MuSCs. P-values were calculated by Mann–Whitney tests comparing control and DMD groups within the same age range

eMHC immunostaining showed that the extent of muscle regeneration diminishes over time, exhibiting a dramatic loss by 9 years with no detected newly formed myofibers (Fig. 3a and b). The DMD del52 rat model, recently described by us [39], shows similar findings, as well as a smaller cohort of DMD muscle biopsies from younger patients. Muscle regeneration relies on muscle stem cell differentiation and self-renewal. We therefore quantified the total number of MuSCs to investigate whether these cells are lost during DMD progression (Fig. 3c). MuSCs were recognized by the expression of Pax7, while to evaluate MuSC activation we used Ki67 as a marker of cell proliferation. We showed that the number of MuSCs was increased in DMD biopsies in reference to the control samples at all ages based on the quantification of the number of Pax7-positive cells (Fig. 3d), suggesting that the MuSCs pool is preserved. Moreover, from 1 to 8 years old, around 10–15% of Pax7-positive MuSCs were cycling, being positive for Ki67. However, after 9 years old, the vast majority of MuSCs were no longer proliferating (Fig. 3e), correlating with the absence of newly regenerated myofibers, suggesting the establishment of quiescence of the muscle stem cell pool (Fig. 3a). We additionally assessed the proportion of senescent MuSCs through the identification of senescence markers, including P16, γH2AX and P21 [19]. Our observations revealed an increased number of MuSCs expressing these senescence markers in DMD muscles compared to controls. By quantifying Pax7-positive cells expressing P16 or γH2AX, we detected a high number of MuSCs expressing these two senescent markers in biopsies from 1–2 years old DMD patients (Fig. 3f and g). Interestingly, the percentage of MuSCs expressing P16 (Fig. 3f) is increasing with the age progression in DMD samples while the percentage of MuSCs expressing P21 (Additional file 1: Fig. S2) or γH2AX (Fig. 3g) remained consistently high over time.

## Discussion

Myopathology has been essential for the diagnosis and for the understanding of the pathophysiological mechanisms of DMD. Myopathologic markers are currently used as readouts for multiple therapies [2, 26]. In this study, we analyzed skeletal muscle biopsy samples from a cohort of DMD and control patients with the aim to delineate the DMD myopathologic trajectory with patients age. We categorized DMD and control samples based on age at muscle biopsy since we observed no notable correlations between the different histopathologic markers examined and the age at first symptoms, genotype or serum Creatine Kinase. Our analysis confirmed that the number of hypercontracted fibers is higher in DMD samples compared to controls and decreases with the age of the patients, while necrotic and fibers with internalized nuclei are increasing. This is in accordance with previous studies reporting a progressive increase of nuclear internalizations and necrotic fibers associated with a decreased presence of hyper-contracted fibers at older ages [9, 29]. Despite the increasing number of necrotic fibers observed with the progression of DMD, the presence of inflammatory cells, identified by CD45, is marked only until the ages of 7–8, and then sharply diminishes at later stages. This observation supports the idea that the inflammatory response plays a crucial role in DMD pathogenesis at early stages of the disease and that anti-inflammatory treatment may be more effective in young patients [31]. In parallel to tissue inflammation, DMD muscle display fatty deposition and fibrosis [17]. We previously demonstrated that fibrosis is the major muscle modification correlating with poor motor outcome in DMD patients [9]. The current study supports the higher abundance of fibrosis within DMD muscles, that become statistically significant by 3–4 years of age compared to control. The elevated fibrotic build-up in DMD muscles did not show statistical significance at 1–2 years, although the level of fibrosis is reaching around 30% of the muscle area in DMD biopsies, suggesting an important fibrotic deposit already at this stage of the disease. This is in line with another study that has shown early-stage fibrosis in DMD samples linked to intrauterine muscle degeneration [29]. Further studies including a higher number of muscle biopsies at perinatal age are needed to elucidate the age of appearance of fibrosis in DMD samples. Interestingly fibrosis did not increase with the age in DMD muscles. This result is in accordance with a previous study from Desguerre et al. [9], showing the absence of a correlation between age at biopsy and fibrosis. On the other side, Peverelli et al. [29] reported a peak of amount of connective tissue at the age of 6–7 years, determining this time period as crucial for the fibrotic degeneration and loss of regeneration. The absence of an increased fibrotic deposition by 7 years in our cohort of DMD patients can be explained by differences in muscle type, sample number, and methods for fibrosis quantification. Indeed, we analyzed fibrosis by quantifying Sirius red area, reflecting collagen deposition, while in the study by Peverelli et al. [29] the fibrosis was assessed by color substraction from hematoxylin and eosin stainings.

Although it is important to consider that the disease progression can vary between individuals, the lack of higher fibrotic deposition with age may be due to tissue adaptation to the chronic injury. Also, being fibrosis a response to inflammation and to the attempts at muscle repair, the plateau in fibrosis may be linked to the lower presence of inflammatory cells or loss of muscle regeneration observed in DMD. Conversely, fat tissue accumulation in DMD muscles is increasing progressively with age, as previously demonstrated [49]. In accordance with this finding, the utilization of qMRI to assess fat replacement is an outcome measure used in clinical trials to determine disease progression [14, 47, 48]. Our results demonstrate that the accumulation of fat tissue in dystrophic muscle continues to increase after the age of ten thus being a more reliable prognostic marker than fibrosis and a potential therapeutic target. The main actors of muscle substitution are muscle mesenchymal progenitors, also known as FAPs [42, 43]. These cells have been associated with the pathogenesis of Duchenne in the animal models of the disease, contributing to both fibrosis substitution and fat tissue accumulation [28, 36, 39, 43]. From our data, the number of FAPs is proportional to fibrotic levels confirming their primary commitment into fibrogenic differentiation. Because fat deposition is increasing over time, we can speculate that the adipogenic potential of FAPs is only occurring at the latest stage of the disease. FAPs also influences muscle tissue regeneration [20, 33]. The ability of the muscle to regenerate relies on muscle stem cells, which after muscle tissue damage, activate and proliferate to create a pool of myoblasts, which eventually differentiate into newly generated myofibers [30, 41]. Previous studies and our study on the DMD rat model del52 showed impaired muscle regeneration [38, 40]. To elucidate the extent of muscle regeneration in our cohort, we quantified the number of regenerating myofibers over time, reporting a dramatic and sustained decrease of newly formed fibers from 7 years old onward. Different hypotheses have been proposed to explain the loss of the regeneration potential in DMD, such as impaired cellular divisions or telomere shortening [11, 12, 32, 46]. Strikingly, we showed a preserved pool of MuSCs in DMD muscles marked by an important loss of cellular activation, suggesting that MuSCs are maintained in DMD muscles, but they lose their proliferation capacity, essential to form a pool of myoblasts committed to the generation of new myofibers. Recently, it has been proposed that loss of muscle repair in DMD is associated with the acquisition of a senescent phenotype of DMD MuSCs [27, 38, 40]. Senescence is a cellular stress response characterized by a stable cell-cycle arrest, resistance to apoptosis and a robust senescence-associated secretory phenotype (SASP) [15]. This acquired condition observed in DMD MuSCs may explain the loss of muscle regeneration with the maintenance of MuSC number and prove that MuSC senescence can be a relevant therapeutic target to promote tissue repair. Indeed, the entry into senescence of certain subsets of muscle stem cells not only decreases the pool of available proliferative myoblasts, but also imparts a state of inflammation that resembles the detrimental effects of inflammation through the secretion of SASP [27]. In line with that, the clearance of senescent MuSCs has been proved to ameliorate the regenerative potential in animal model of DMD [27, 38]. Also, pharmacological prevention of DMD MuSC senescence entry improves muscle regeneration [40]. These recent findings together with our longitudinal study of MuSC senescence in twenty-four DMD muscle biopsies further support the clinical significance of preventing senescence in DMD muscle stem cells. We confirmed that DMD MuSCs acquire an early senescent phenotype, using three different markers of cellular senescence in vivo (P16, γH2AX and P21). While γH2AX and P21 expression levels are constantly maintained in DMD muscle stem cells across different ages, the number of P16-positive MuSCs is increasing with the disease progression. We, thus, propose P16 as a useful biomarker to analyze muscle repair loss and MuSC senescence in DMD muscles. In summary, our work has an important descriptive relevance in characterizing the typical hallmarks of DMD, as fiber damage, fibrosis, fatty deposition and inflammation, as well as novel features of the disease like FAP accumulation, loss of muscle regeneration and MuSC senescence in a cohort of DMD patients ranging from 1 to 18 years of age.

## Conclusions

This study has assembled one of the largest cohorts of muscle biopsies of DMD patients, which are currently extremely rare because DMD diagnosis is based primarily on genetics. Our analyses allowed to get valuable insights into the myopathologic trajectory of DMD hallmarks and muscle regeneration impairment with concurrent senescence entry of muscle stem cells alongside with accumulation of fibro-adipogenic progenitors in relation with fibrosis and fat accumulation. These findings hold a high relevance for pharmacological trials that employ muscle biopsy modifications as outcome measures.

### Supplementary Information

**Additional file 1** Fig.S1. Dystrophin immunostaining **A**) Immunofluorescence for Dystrophin (red) on control (CTR) and DMD biopsies. Scale bar=20μm; Fig. S2. P21 expression in MuSCs. **A**) Quantifications of Pax7-positive MuSCs expressing P21on control (CTR) and DMD biopsies.

## Data Availability

All data are present in the paper and available via material transfer agreement by contacting the corresponding author.

## Abbreviations

DMD: Duchenne muscular dystrophy
FAPs: Fibro-adipogenic progenitors
MuSCs: Muscle stem cells
Nr: Not reported
Q: Quadriceps
D: Deltoid
Do: Dorsal muscles
