# Supplementary material for: Myopathologic trajectory in Duchenne muscular dystrophy (DMD) reveals lack of regeneration due to senescence in satellite cells
Source: Acta Neuropathol Commun. 2023 Oct 19;11:167. doi: 10.1186/s40478-023-01657-z (PMC10585739; doi:10.1186/s40478-023-01657-z)
Supplement: Supplementary file 1 — Additional file 1 Fig.S1. Dystrophin immunostaining A) Immunofluorescence for Dystrophin (red) on control (CTR) and DMD biopsies. Scale bar=20μm; Fig. S2. P21 expression in MuSCs. A) Quantifications of Pax7-positive MuSCs expressing P21on control (CTR) and DMD biopsies. [file 40478_2023_1657_MOESM1_ESM.pdf]

Myopathologic trajectory in Duchenne Muscular Dystrophy (DMD) reveals lack of regeneration due to senescence in satellite cells

Acta Neuropathologica Communications

Nastasia Cardone PhD[1#], Valentina Taglietti PhD[1#], Serena Baratto MSc[2], Kaouthar Kefi BSc[1], Baptiste Periou BSc[1,3], Ciryl Gitiaux MD PhD[4,5], Christine Barnerias MD[5], Peggy Lafuste PhD[1], France Leturcq Pharm[6], Juliette Nectoux Pharm, PhD[6], Chiara Panicucci MD PhD[2], Isabelle Desguerre MD PhD[4], Claudio Bruno MD, PhD[2,7], François-Jerome Authier MD PhD[1,4], Chiara Fiorillo MD PhD[7,8], Frederic Relaix PhD[1,4*°], Edoardo Malfatti MD, PhD[1,4*°]

[1] Univ Paris Est Creteil, INSERM, IMRB, F-94010 Creteil, France
[2] Centre of Translational and Experimental Myology, IRCCS Istituto Giannina Gaslini, Genova, Italy
[3] APHP, Filnemus, EuroNMD, Centre de Référence de Pathologie Neuromusculaire Nord-Est-Ile-de-France, Henri Mondor Hospital, France
[4] Neurophysiologie clinique pédiatrique, Centre de référence des maladies neuromusculaires Hôpital universitaire Necker-Enfants Malades-Paris
[5] Reference Center for Neuromuscular Disorders, Filnemus, EuroNMD, Assistance Publique-Hôpitaux de Paris (APHP) Necker Enfants Malades Hospital, Paris, France.
[6] Service de Médecine Génomique, Maladies de Système et d'Organe - Fédération de Génétique et de Médecine Génomique, DMU BioPhyGen, APHP Centre-Université Paris Cité - Hôpital Cochin, Paris, France [7]Department of Neuroscience, Rehabilitation, Ophthalmology, Genetics, Maternal and Child Health-DINOGMI, University of Genova,
[8]Child Neuropsychiatry, IRCCS Istituto Giannina Gaslini, Genova, Italy

#Co-first Authors
*Co-last Authors
°Co-Corresponding Author
edoardo.malfatti@aphp.fr

Supplementary Information (SI)

Fig. S1

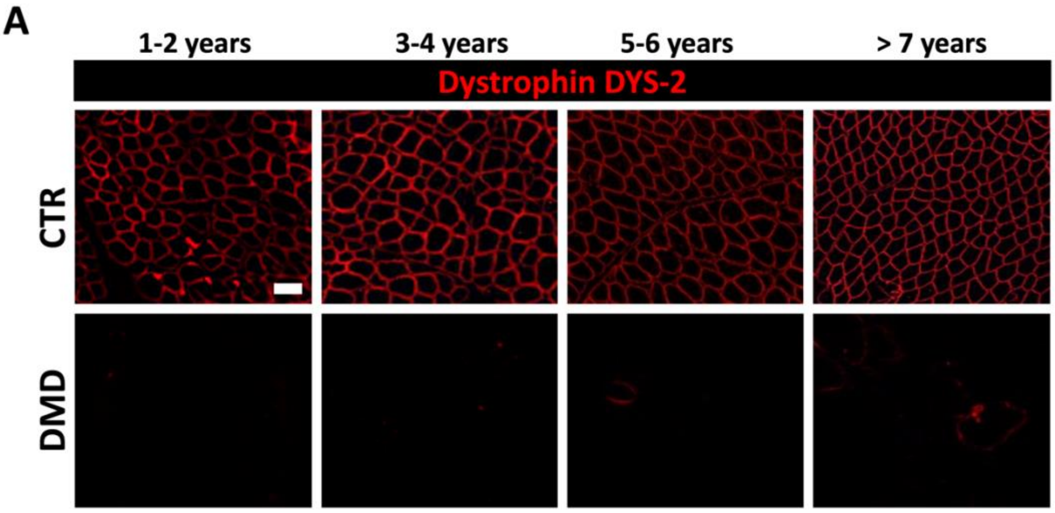

Fig.S1. Dystrophin immunostaining

A) Immunofluorescence for Dystrophin (red) on control (CTR) and DMD biopsies. Scale bar=20μm;

Fig. S2

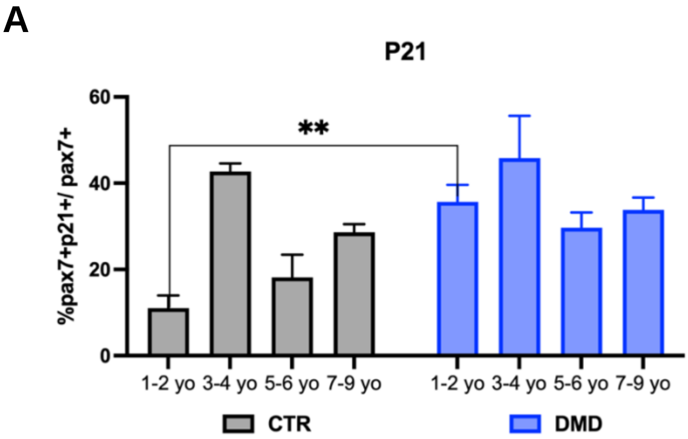

Fig. S2. P21 expression in MuSCs.

A) Quantifications of Pax7-positive MuSCs expressing P21on control (CTR) and DMD biopsies.
